# The Shc Family Protein Adaptor, Rai, Negatively Regulates T Cell Antigen Receptor Signaling by Inhibiting ZAP-70 Recruitment and Activation

**DOI:** 10.1371/journal.pone.0029899

**Published:** 2011-12-29

**Authors:** Micol Ferro, Maria Teresa Savino, Barbara Ortensi, Francesca Finetti, Luca Genovese, Giulia Masi, Cristina Ulivieri, Daniela Benati, Giuliana Pelicci, Cosima T. Baldari

**Affiliations:** 1 Department of Evolutionary Biology European Institute of Oncology, Milan, Italy; 2 Department of Experimental Oncology, European Institute of Oncology, Milan, Italy; 3 Istituto Toscano Tumori, University of Siena, Siena, Italy; Oklahoma Medical Research Foundation, United States of America

## Abstract

Rai/ShcC is a member of the Shc family of protein adaptors expressed with the highest abundance in the central nervous system, where it exerts a protective function by coupling neurotrophic receptors to the PI3K/Akt survival pathway. Rai is also expressed, albeit at lower levels, in other cell types, including T and B lymphocytes. We have previously reported that in these cells Rai attenuates antigen receptor signaling, thereby impairing not only cell proliferation but also, opposite to neurons, cell survival. Here we have addressed the mechanism underlying the inhibitory activity of Rai on TCR signaling. We show that Rai interferes with the TCR signaling cascade one of the earliest steps –recruitment of the initiating kinase ZAP-70 to the phosphorylated subunit of the TCR/CD3 complex, which results in a generalized dampening of the downstream signaling events. The inhibitory activity of Rai is associated to its inducible recruitment to phosphorylated CD3, which occurs in the physiological signaling context of the immune synapse. Rai is moreover found as a pre-assembled complex with ZAP-70 and also constitutively interacts with the regulatory p85 subunit of PI3K, similar to neuronal cells, notwithstanding the opposite biological outcome, i.e. impairment of PI-3K/Akt activation. The data highlight the ability of Rai to establish interactions with the TCR and key signaling mediators which, either directly (e.g. by inhibiting ZAP-70 recruitment to the TCR or sequestering ZAP-70/PI3K in the cytosol) or indirectly (e.g. by promoting the recruitment of effectors responsible for signal extinction) prevent full triggering of the TCR signaling cascade.

## Introduction

Rai, also known as N-Shc/ShcC, belongs to the Shc family of protein adaptors. This family includes four members which collectively act as central participants in the signaling pathways triggered by tyrosine kinase-coupled surface receptors controlling a number of cellular processes, including proliferation, differentiation, survival and motility [1]. As all Shc proteins, Rai has a modular structure characterized by a central collagen homology (CH1) domain containing five phosphorylatable tyrosine residues flanked by a N-terminal PTB domain and a C-terminal SH2 domain [2], [3].

*Rai* encodes two proteins, of 52 and 64 kDa respectively, which are present at high levels in the CNS, with a selective expression in post-mitotic and mature neurons. Expression of Rai in the CNS is developmentally regulated, with a pattern opposite to ShcA. During embryonic development Rai is absent in developing neurons, where ShcA is instead highly expressed and promotes the proliferation of neuronal stem cells. As neuronal progenitors differentiate ShcA is progressively downregulated and replaced by Rai, which reaches maximal levels in the adult brain [2]–[5].

Rai expression has been associated with differentiation and survival of neuronal cells, where it couples tyrosine kinase receptors such as Ret to the PI-3K/Akt survival pathway [6]. Rai promotes moreover sustained activation of MAP kinases, thereby contributing to neuronal differentiation [6], [7]. Of note, at variance with ShcA, the latter activity does not involve recruitment of Grb2/Sos complexes, as none of the phoshorylatable tyrosine residues in the CH1 domain is a high affinity binding site for Grb2 [7]. The PI-3K/Akt dependent pro-survival function of Rai has also been established in the pathological context of hypoxia and oxidative stress, as demonstrated by the enhanced apoptotic response of cortical neurons from Rai^−/−^ mice and the more severe neurological damage and size of infarct area in a model of brain ischemia/reperfusion injury [8]. More recently, Rai has been implicated in retinal development and regeneration [9]. Moreover, ectopic or abnormal Rai expression has been associated to some types of cancer, including aggressive neuroblastomas and thyroid carcinomas [10]–[12].

While expression of Rai is restricted prevalently to neurons, Rai is also expressed, albeit at lower levels, in other cell types, such as enteric glial cells, endothelial cells and smooth muscle cells of the gastrointestinal tract [13], indicating potential functions of this adaptor outside of the CNS. In support of this notion, we found that the p52 kDa isoform of Rai is expressed in both T and B lymphocytes. Interestingly, in these cells Rai antagonizes activation and survival pathways triggered by the antigen receptors, at variance with its function in neurons. Rai^−/−^ mice display pathological features consistent with this inhibitory function, including splenomegaly, spontaneous T- and B-cell activation and autoantibody production, which eventually lead to the development of a lupus-like autoimmune disease [14].

While a function of Rai as a negative regulator of TCR and BCR signaling has clearly emerged from this study, the mechanism by which Rai modulates the respective signaling cascades has as yet not been elucidated. Here we demonstrate that Rai inhibits TCR signaling at one of the earliest steps in the cascade, recruitment of ZAP-70 to the activated receptor, thereby preventing productive downstream signaling.

## Results

### Rai dampens TCR signaling

To elucidate the mechanism underlying the inhibitory activity of Rai on TCR signaling we initially used Jurkat T cells, which do not express Rai at detectable levels, to generate a transfectant stably expressing the p52 isoform of Rai. A Jurkat line stably transfected with empty vector was used as control. Given the partial homology of the PTB and SH2 domains among Shc family members, as control of specificity we also generated similar transfectants using as recipient the Jurkat variant JSL1, which is deficient for ShcA expression [15]. The immunoblot analysis of Rai expression in the Jurkat and JSL1 transfectants is shown in figure S1A.

**Figure 1:**
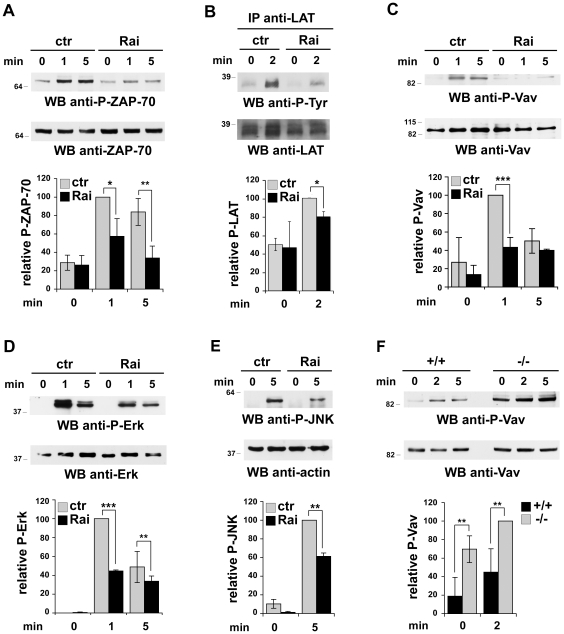
Rai dampens TCR signaling. **A–E**. Immunoblot analysis of ZAP-70 (**A**), LAT (**B**), Vav (**C**), Erk1/2 (**D**) and JNK (**E**) phosphorylation in postnuclear supernatants from control and Rai-expressing Jurkat transfectants activated with anti-CD3 mAb for the indicated times. Phosphospecific antibodies were used on total postnuclear supernatants to analyze the activation of ZAP-70, Vav, Erk and JNK, while the activation of LAT was analyzed by immunoblot of LAT specific immunoprecipitates with anti-phosphotyrosine antibodies. The quantification by laser densitometry of the relative levels of phosphorylation of each of the proteins is shown below the representative immunoblots (*n*≥3; *n* = 2 for P-JNK). **F**. Immunoblot analysis of Vav phosphorylation in postnuclear supernatants from splenocytes from wild-type (+/+) or Rai^−/−^ (−/−) mice activated with anti-CD3 mAb. The quantification by laser densitometry of the relative levels of Vav phosphorylation is shown below the representative immunoblot (*n*≥3). ***p≤0.001, **p≤0.01*p≤0.05.

The impact of Rai expression on TCR signaling was assessed by immunoblot analysis of cell lysates with phosphospecific antibodies which recognize the active forms of ZAP-70, Vav, Erk and JNK, respectively. The activation of LAT was moreover assessed by immunoblot analysis of LAT-specific immunoprecipitates with anti-phosphotyrosine antibodies. As shown in figure 1A, activation of ZAP-70, one of the earliest events in the TCR signaling cascade, was significantly lower in Rai-expressing Jurkat cells compared to control cells. In agreement with this result, Rai expression resulted in impaired activation of the transmembrane adaptor LAT (Fig. 1B), which couples the TCR to multiple signaling pathways [16], as well as of Vav (Fig. 1C), a Rho family specific guanine nucleotide exchanger which regulates the reorganization of cortical actin required for the topological orchestration of the TCR signaling cascade during immune synapse assembly [17]. Moreover, consistent with the defect in LAT and Vav phosphorylation, activation of Erk and JNK, which couple the TCR to gene transcription downstream of Ras and Rho GTPases, respectively [18], [19], was found to be blunted in the presence of Rai (Fig. 1D,E). In agreement with these findings and with our previous report showing that Rai deficiency potentiates TCR dependent activation of ZAP-70 and the MAP kinases in mouse T cells [14], Vav phosphorylation was significantly enhanced both in the absence of stimulation and in response to TCR triggering in splenic T cells from Rai^−/−^ mice compared to their wild-type counterparts (Fig. 1F). Hence Rai acts as a negative regulator of TCR signaling beginning from an early step in the cascade, i.e. ZAP-70 activation.

### Rai impairs TCR dependent ZAP-70 activation by interfering with its recruitment to phosphorylated CD3

**Figure 2:**
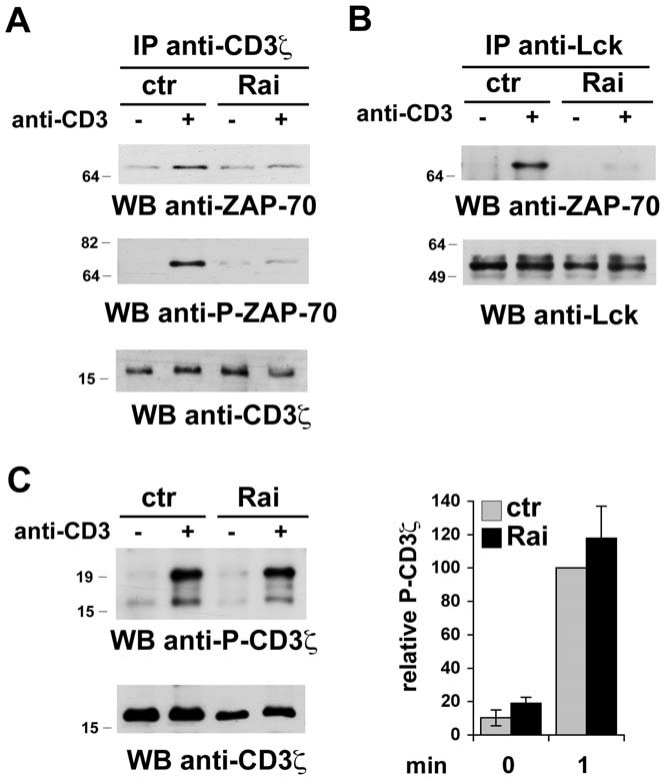
Rai interferes with ZAP-70 recruitment to phosphorylated CD3. **A,B**. Immunoblot analysis with anti-ZAP-70 or anti-phospho-ZAP-70 antibodies of CD3- (**A**) or Lck- (**B**) specific immunoprecipitates from post-nuclear supernatants of the control and Rai-expressing Jurkat transfectants activated with anti-CD3 mAb for 15 sec. Control blots of the stripped filters are shown below. **C**. Immunoblot analysis with anti-phospho-CD3 mAb and respective control blot of postnuclear supernatants from the control and Rai-expressing Jurkat transfectants activated for 1.5 min. The histogram shows the quantification by laser densitometry of the relative levels of CD3 phosphorylation (*n*>3).

ZAP-70 activation is initiated by its recruitment to the phosphorylated ITAMs of the CD3 chain. Full activation requires its interaction with and phosphorylation by the tyrosine kinase Lck [20]. To elucidate the mechanisms underlying the inhibition of TCR dependent ZAP-70 activation by Rai, the interaction of ZAP-70 with CD3was analyzed in co-immunoprecipitation experiments in the Jurkat T cell transfectants. As opposed to control cells, where the basal interaction of ZAP-70 with CD3was enhanced in response to TCR engagement, no significant increase was observed in Rai-expressing cells (Fig. 2A). Moreover, immunoblot analysis with phosphospecific antibodies showed that the small amount of CD3-bound ZAP-70 was phosphorylated to barely detectable levels compared to control cells (Fig. 2A). The interaction of ZAP-70 with Lck was also dramatically impaired in Rai-expressing cells (Fig. 2B), consistent with the fact that this event occurs after ZAP-70 recruitment to the activated TCR. On the other hand, TCR dependent CD3 phosphorylation was comparable in control and Rai-expressing cells (Fig. 2C). Collectively, these results indicate that Rai acts downstream of CD3 phosphorylation by interfering with ZAP-70 recruitment to the activated TCR, which results in impaired ZAP-70 activation.

### Rai is phosphorylated in response to TCR engagement and interacts with CD3 and ZAP-70

**Figure 3:**
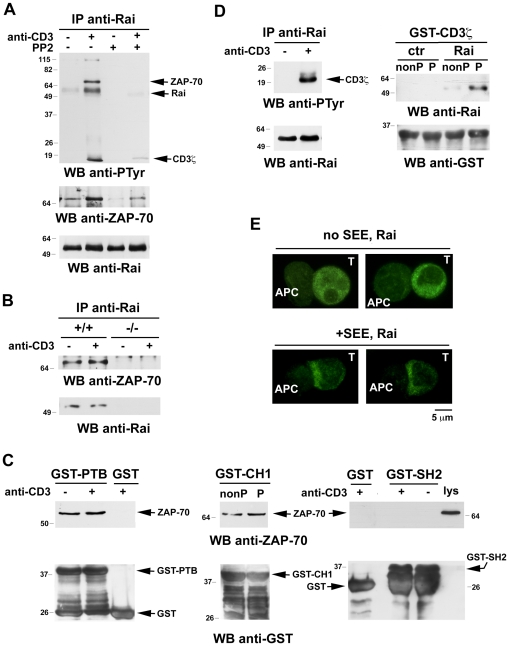
Rai forms a complex with CD3 and ZAP-70 and clusters at the immunological synapse. **A**. Immunoblot analysis with anti-phospho-tyrosine or anti-ZAP-70 antibodies of Rai-specific immunoprecipitates from post-nuclear supernatants of the Rai-expressing Jurkat transfectant activated with anti-CD3 mAb for 1.5 min. Where indicated cells were incubated with 20 µM PP2 prior to activation. A control blot of the stripped filter is shown below. **B**. Immunoblot analysis with anti-ZAP-70 antibodies of Rai-specific immunoprecipitates from post-nuclear supernatants of splenocytes from wild-type (+/+) or Rai^−/−^ (−/−) mice activated with anti-CD3 mAb for 1.5 min. A control blot is shown below. **C**. Immunoblot analysis with anti-ZAP-70 antibodies of *in vitro* binding assays carried out on post-nuclear supernatants of the control Jurkat line using GST fusion proteins containing the PTB, SH2 or CH1 domain of Rai, the latter either non-phosphorylated or phosphorylated *in vitro* with recombinant Lck. Control blots with anti-GST antibodies are shown below. **D**. *Left*, Immunoblot analysis with anti-phospho-tyrosine antibodies of Rai-specific immunoprecipitates from post-nuclear supernatants of the Rai-expressing Jurkat transfectant activated with anti-CD3 mAb for 1.5 min. *Right*, Immunoblot analysis with anti-Rai antibodies of *in vitro* binding assays carried out on post-nuclear supernatants of control or Rai-expressing Jurkat cells using a GST fusion protein containing the intracellular domain of human CD3, either non-phosphorylated or phosphorylated *in vitro* with recombinant Lck. A control blot of the stripped filter is shown below. **E**. Immunofluorescence analysis of Rai in conjugates of Rai-expressing Jurkat cells and antigen-pulsed APC (SEE), incubated at 37°C for 15 min. Conjugates formed in the absence of antigen are shown on top part of the panel. Representative images are shown. No aspecific fluorescence was observed when Rai-expressing cells were immunostained with secondary antibody alone (not shown).

The modular structure of Rai, together with its phosphorylatable tyrosine residues, suggests that Rai may interfere with ZAP-70 recruitment to phospho-CD3 through the formation of inhibitory complexes. To address this point Rai-specific immunoprecipitates were analyzed by immunoblot, using anti-phosphotyrosine antibodies. As shown in figure 3A, Rai was found to be phosphorylated on tyrosine residues in response to TCR engagement. No phosphorylation was observed when cells were pretreated with the Lck inhibitor, PP2, indicating that Rai is either a direct substrate of Lck or the substrate of a kinase, such as ZAP-70, the activation of which requires Lck.

Three major phosphoproteins, of approximately 20, 70 and 115 kDa, were found to inducibly associate with Rai in response to TCR engagement. Reprobing the stripped filters with anti-ZAP-70 antibodies showed that the 70 kDa phosphoprotein corresponds to ZAP-70. Interestingly, Rai also interacts with non-phosphorylated ZAP-70. This interaction was detectable both in the absence of stimulation and in PP2-treated cells, albeit at a reduced extent under these conditions, indicating that it is at least in part phosphorylation-independent (Fig. 3A). TCR triggering resulted in an enhancement in ZAP-70 binding to Rai (Fig. 3A). These results were confirmed in the Rai-expressing JSL1 transfectant (Fig.S1B). Consistent with these data, Rai was found to constitutively interact with ZAP-70 in T cells from wild-type mice. This interaction was enhanced in response to TCR engagement (Fig. 3B). Pull-down assays using the isolated domains of Rai showed that ZAP-70 strongly interacts with the PTB domain independently of TCR engagement (Fig. 3C), suggesting that this domain is responsible to a major extent for the constitutive association of Rai with ZAP-70. A basal interaction of ZAP-70 was also observed with the CH1 domain of Rai. This interaction was however enhanced when the CH1 domain was phosphorylated in vitro using recombinant Lck, suggesting that the increase in Rai association with ZAP-70 triggered by TCR engagement is mediated by the phosphorylated tyrosine residues in the CH1 domain. At variance, the SH2 domain of Rai failed to pull down detectable amounts of ZAP-70 (Fig. 3C).

Based on its electrophoretic mobility, the approximately 20 kDa phosphoprotein which associates with Rai in the activated Jurkat transfectant (Fig. 3A,D) is likely tyrosine phosphorylated CD3. This interaction was confirmed in *in vitro* binding experiments on lysates of non-stimulated cells, using a GST-CD3 fusion, either non-phosphorylated or phosphorylated *in vitro* with recombinant Lck (Fig. 3D).

### Rai is recruited to the immune synapse

Signaling by the TCR is topologically orchestrated at the immune synapse. This dynamic interface, that forms when the T cell encounters an APC carrying cognate antigen, coordinates positive and negative signals triggered by TCR, CD4/CD8, costimulatory receptors and integrins engaged by their respective ligands on the surface of the APC [21]. To understand whether the association of Rai with CD3 observed biochemically occurs in the context of immune synapse assembly, Rai was visualized by immunofluorescence on antigen specific conjugates formed between Rai-expressing Jurkat cells and Raji B cells loaded with staphylococcal enterotoxin E (SEE). Conjugates formed in the absence of SEE were used as controls. As shown in figure 3E (top), Rai showed a diffuse distribution in control conjugates, consistent with its cytosolic localization. At variance, in the presence of SEE a significant enrichment in Rai could be observed at the contact area with the APC (Fig. 3E, bottom). Hence Rai is recruited to the immune synapse, where it can associate with phosphorylated CD3.

### Rai interacts with p85 PI-3K

**Figure 4:**
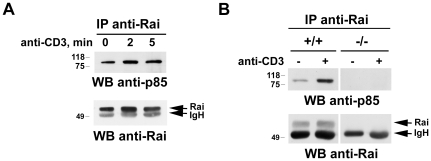
Rai interacts with p85 PI3K. **A,B**. Immunoblot analysis with anti-p85 antibodies of Rai-specific immunoprecipitates from post-nuclear supernatants of the Rai-expressing Jurkat transfectant (**A**) or of splenocytes from wild-type (+/+) or Rai^−/−^ (−/−) mice (**B**), activated with anti-CD3 mAb for the indicated times. Control blots of the stripped filters are shown below.

We have previously reported that Rai deficiency in the mouse results not only in enhanced mitogenic signaling by the TCR, but also in enhanced survival signaling [14], as assessed by activation of the pro-survival kinase, Akt, one of the major targets of PI-3K [22]. Based on the known interaction of Rai with p85, the regulatory subunit of PI-3K, in neuronal cells [6], we addressed their potential association in T cells. Immunoblot analysis of Rai-specific immunoprecipitates showed an interaction of p85 with Rai in the absence of stimulation, which was modestly enhanced in response to TCR engagement (Fig. 4A; FigureS1C for the Rai-expressing JSL1 transfectant). This result was confirmed on splenic mouse T cells, where the Rai/p85 association was however clearly potentiated in response to TCR triggering (Fig. 4B). Taken together with the inhibitory effect of Rai on Akt activation [14] the results indicate that, although Rai interacts with p85, similar to neuronal cells, this interaction is not functional to the activation of PI-3K.

## Discussion

While the central function of molecular adapters in coupling cell surface receptors to multiple intracellular signaling is largely established, it is only in recent years that their role as negative regulators has emerged. The mechanisms by which adapters subserve this function are remarkably diverse. In T cells adapters such as PAG and SLAP prevent initiation of TCR signaling by inhibiting the activation of the initiating kinase Lck and by promoting the degradation of recycling TCR complexes, respectively [23], [24]. Others, such as LAT and TRIM, are implicated in signal extinction by establishing feedback loops through the recruitment of phosphatases, and by increasing the levels of surface CTLA-4, respectively [16], [23]. The majority of the adaptors with a negative regulatory function act however as dampeners of TCR signaling. These include LAX, NTAL and the Dok family proteins, Dok1, Dok2 and Dok4, as well as p66Shc [1], [16], [25]. Interestingly, while in cell models specific steps in TCR signaling appear to be modulated by individual adapters (e.g. Ras activation through RasGAP recruitment by Dok1/2, PI3K activation by LAX), in many instances T cells from mice genetically deficient for these molecules display a generalized hyper-reactivity to TCR engagement beginning from the earliest steps, as documented for LAX, NTAL, Dok1/2 and p66Shc deficient T cells [26]–[29]. Hence the precise mechanisms by which these adaptors dampen TCR signaling is a still open question.

Similar to the latter adaptors, Rai appears to act as a general dampener of antigen receptor signaling. As previously reported, Rai deficiency in the mouse results in a hyper-reactivity of peripheral T and B cells to antigen receptor engagement both *in vitro* and *in vivo*, which eventually leads to the development of lupus-like autoimmunity [14]. Specifically, this study provided evidence that Rai modulates the TCR signaling cascade as early as ZAP-70 and PI-3K activation, which results in impaired Erk and Akt activation. Here we have addressed the underlying mechanism. The results show that Rai is recruited to phosphorylated CD3 in response to TCR engagement. The relevance of this association is underlined by the accumulation of Rai at the immune synapse. The interaction of Rai with CD3 is dependent on CD3 phosphorylation, which is not affected by the presence of Rai. Under these conditions ZAP-70 fails to be effectively recruited to the TCR, which results in its defective interaction with Lck and impaired activation. This suggests that Rai may limit the access of ZAP-70 to phosphorylated CD3, as suggested for SLAP [30], or alternatively induce its release from the TCR by establishing an inhibitory interaction. This would provide an explanation as to why at least part of the ZAP-70 molecules that interact with Rai are phosphorylated following TCR engagement, despite the inhibitory activity of Rai on TCR signaling. Importantly, the pool of Rai participating in a pre-assembled complex with ZAP-70 in quiescent cells is unlikely to participate in the interaction with the TCR, as one would expect to observe an increase in CD3-bound ZAP-70 in response to TCR engagement, which is not the case. In this scenario, Rai would act as a sink to sequester ZAP-70 in a state incompetent to associate with CD3. Alternatively (or additionally), similar to other adaptors that dampen TCR signaling or establish negative feedback loops, Rai may exploit its adaptor function to recruit close to the activated TCR molecules implicated in signal termination, such as protein tyrosine phosphates (e.g. SHP-2), lipid phosphatases (e.g. SHIP-1) or ubiquitin ligases (e.g. c-Cbl). Although their molecular mass made them potential candidates to the approximately 115 kDa phosphoprotein that coprecipitates with Rai in response to TCR engagement (Fig. 3), we did not detect any association of Rai with either c-Cbl or SHIP-1, nor did we observe an interaction with SHP-2 (our unpublished results).

In addition to its constitutive interaction with ZAP-70, Rai is also involved in a constitutive, phosphotyrosine independent interaction with p85, the regulatory subunit of type I PI-3K. A similar interaction occurs in neuronal cells, where Rai couples the Ret receptor to PI-3K activation by forming a pre-assembled complex with Gab1 and p85, which is recruited to the activated receptor following ligand binding [12]. The functional outcome of the association of Rai with p85 is however dramatically different in neuronal cells and T cells. Recruitment of the Rai/p85 complex to Ret results in activation of the Akt dependent survival pathway, an activity which has been confirmed in the *in vivo* setting of Rai^−/−^ mice [14]. Conversely, we have previously reported that TCR dependent Akt activation is enhanced in Rai^−/−^ T cells, which correlates with increased cell survival [14]. This inhibitory activity of Rai on cell survival extends to B cells [14]. Hence Rai promotes lymphocyte survival in response to antigen receptor signaling, as opposed to neuronal cells, indicating that its interaction with p85 is not functional to the activation of p110, the catalytic subunit of PI3K. Whether, similar to what has been proposed for the adapter LAX [26], Rai limits PI-3K activation by sequestering p85 remains to be determined. An alternative possibility is that the attenuation in TCR dependent PI-3K/Akt activation observed in the presence of Rai is independent of its association with p85 and rather reflects a generalized defect in TCR signaling resulting from the inhibitory effect of Rai on the key early event, ZAP-70 activation. The fact that Rai is implicated in opposite biological outcomes in different cell types is intriguing but not unique. A similar dual function has been described for example for NTAL, which acts as a positive regulator of BCR signaling and a negative regulator of TCR signaling [27], [31]. Obviously the cellular context is likely to be determinant by providing binding partners that, depending on their specific function, will dictate the final biological outcome.

To clearly delineate the mechanism of attenuation of TCR signaling by Rai the interplay with other Shc proteins expressed in T cells will eventually have to be taken into account. Among these, all three ShcA isoforms are present in T cells, with the abundantly expressed p52Shc isoform coupling the TCR to the Ras and Rho small GTPases and the less abundant p66Shc isoform dampening TCR signaling, similar to Rai. Whether Rai cooperates with p66Shc in this function remains to be understood. Nevertheless, the function of Rai appears not to be redundant, as shown by the immune alterations in Rai^−/−^ mice. This notion is further supported by the differences in the pathological features in mice lacking Rai and p66Shc, which include membranous autoimmune glomerulonephritis and alopecia as unique features of p66Shc^−/−^ mice [29]. Another important consideration is that, while both Rai and p66Shc attenuate TCR signaling by intefering with the initial steps of the cascade, it cannot be ruled out at this stage that individual signaling mediators might be differentially modulated by the two adapters. Specific components of the TCR signaling cascade have been selectively implicated in commitment of CD4^+^ T cell to develop to a specific helper subset, as shown for Akt1 and Vav1 in the development Th1 and Th2 effectors, respectively [32], [33]. Addressing the impact of Rai and p66Shc deficiency on helper T cell polarization may provide insight into the complex process that underlies this fundamental developmental decision.

## Materials and Methods

### Cells and mice

Cell lines included the human T-lymphoma Jurkat line [34], the Shc-deficient Jurkat variant JSL1 [15] and the human Raji B cell line [35]. Jurkat and Raji cells were a generous gift of Dr. D. Boraschi (National Research Council, Pisa, Italy). Stable Jurkat and JSL-1 transfectants were generated using either an expression construct encoding p52Rai or empty vector.

4–6 months old Rai^−/−^ mice backcrossed in C57BL/6, as well as control age- and sex-matched C57BL/6 mice (Harlan, Italia), were used in this study. All mice were maintained in the animal facility of the University of Siena (Siena, Italy) and the IEO-IFOM (Milan, Italy). Mice were housed in a light (07:00 to 19:00) and temperature (18°C–22°C) controlled environment, and food (Global diet 2018; Mucedola, Settimo Milanese, Italy) and water were provided for consumption *ad libitium*. All animal experiments were carried out in agreement with the Guiding Principles for Research Involving Animals and Human Beings and approved by the local ethical committee of the University of Siena, Italy (protocol approval 31 March 2008). Mice were sacrified by cervical dislocation, and all efforts were made to minimize suffering.

### Antibodies and reagents, GST fusions

Phosphospecific antibodies recognizing the active forms of ZAP-70, Erk1/2 and JNK were from Cell Signaling Technology; anti-phospho-CD3ζ from Santa Cruz Biotechnology (Santa Cruz), anti-phospho-Vav from Biosource (Camarillo). The anti-Rai CH1 mAb was from Becton-Dickinson Biosciences; mAbs against Erk2 and CD3ζ, as well as anti-LAT polyclonal antibodies, were from Santa Cruz Biotechnology; anti-phosphotyrosine, anti-Vav, anti-ZAP-70 and anti-LAT mAbs, as well as anti-Lck and anti-Shc polyclonal antibodies, from Upstate Biotechnology Inc; the anti-actin mAb from Millipore. IgG antibodies from OKT3 (mouse anti-human) and 145-2c11 (hamster anti-mouse CD3) hybridoma supernatants were purified on Mabtrap (Amersham Biosciences, GE Healthcare Life Sciences Italia) and titrated by flow cytometry. Both hybridomas were obtained from the Americal Type Culture Collection (Manassas, USA). PP2 was purchased from Calbiochem (Merck KGaA, Darmstadt, Germany).

The GST fusion containing the cytosolic domain of CD3 was previously described [36]. The cDNAs encoding the PTB, CH1 and SH2 domains of human Rai were inserted into the bacterial expression plasmid pGEX6P. The recombinant fusion proteins, as well as control GST, were affinity purified on GSH-Sepharose (GE Healthcare) from bacterial cultures incubated for 4 h at 37°C with 0.25 mM isopropyl-β-D-thiogalactopyranoside and lysed by sonication in PBS-1% Triton X-100.

### Cell activation and lysis, immunoprecipitations, immunoblots and *in vitro* binding assays

Jurkat transfectants (5×10^6^ cells/sample) or splenocytes from control or Rai^−/−^ mice (10×10^6^/sample) were activated by cross-linking the TCR/CD3 complex in solution at 37°C using anti-CD3 mAb and secondary antibodies. Cells were lysed in 3% or 1% (v/v) Triton X-100 in 20 mM Tris-HCl (pH 8), 150 mM NaCl (in the presence of 0.2 mg Na orthovanadate/ml, 1 µg/ml pepstatin, leupeptin and aprotinin, and 10 mM phenylmethylsulfonyl fluoride). Postnuclear supernatants were resolved by SDS-PAGE and transferred to nitrocellulose. Alternatively, postnuclear supernatants from 50–100×10^6^ cells/sample were immunoprecipitated using the appropriate polyclonal or monoclonal antibodies and protein A Sepharose (GE Healthcare) or anti-mouse IgG (whole molecule)-Agarose (Sigma Aldrich, Milan, Italy), respectively. Immunoblots were carried out using peroxidase-labeled secondary antibodies (GE Healthcare) and a chemiluminescence detection kit (Bio-rad Laboratories Inc, Milan, Italy). Immunoblots were scanned using a laser densitometer (Duoscan T2500) and quantified using the ImageQuant 5.0 software (GE Healthcare). Pre-stained molecular weight markers were purchased from Lonza Milano srl (Italy).

*In vitro*-binding assays were carried using the GST-tagged proteins as described [36] on GSH-Sepharose precleared postnuclear supernatants from 1×10^7^ cells/sample, either activated or not and lysed in 1% Triton X-100 in the presence of protease inhibitors. Where required, GST-CH1 (Rai) and GST-CD3ζ were previously phosphorylated *in vitro* in 20 µl of 20 mM Tris-HCl (pH 7.4), 10 mM MgCl_2_, 10 mM MnCl_2_, 50 µM ATP for 20 min using recombinant Lck (EMD Millipore, Merck KgaA, Darmstadt, Germany). The effectiveness of phosphorylation was checked by immunoblot with anti-phosphotyrosine antibodies.

### Immunofluorescence microscopy

Control or p52Rai-expressing Jurkat cells (1.5×10^5^ cells/4 µl PBS) were fixed with methanol and processed for immunofluorescence microscopy as previously described [37]. For the immune synapse experiments, Raji cells (used as APCs) were pulsed for 2 h with 10 µg/ml SEE and labeled with 10 µM Cell Tracker Blue for 20 min. APC were washed, mixed with Jurkat cells (1∶1) for 15 min and plated on polylysine-coated wells of diagnostic microscope slides (Erie Scientific Company, Portsmouth, USA). Cells were allowed to adhere for 15 min and then processed as described [37]. Antigen-independent conjugates were obtained by mixing T cells and APCs in the absence of SEE. Following fixation, samples were washed 5 min in PBS and incubated for 1 h at RT with anti-Rai mAb. After washing in PBS, samples were incubated for 1 h at RT with Alexa Fluor 488-labeled secondary antibodies. Confocal microscopy was carried out on a Zeiss LSM700 using a 63× objective. Z series of optical sections were performed at 0.5 µm increments. Images were acquired with pinholes opened to obtain optical sections of 0.6 µm thick. Detectors were set to detect an optimal signal below the saturation limits. Images were processed with Zen 2009 image software (Carl Zeiss, Jena, Germany).

### Statistical analyses

Values of specified groups were compared using the Student *t* test statistics, unpaired, provided by the Excel application. A level of p<0.05 was considered statistically significant.

## Supporting Information

Figure S1 **A.** Immunoblot analysis with anti-Rai, anti-Shc and anti-actin antibodies of postnuclear supernatants from Jurkat and JSL1 cells stably transfected with either empty vector (ctr) or the same vector encoding the p52 kDa isoform of Rai. **B,C.** Immunoblot analysis with anti-ZAP-70 (**B**) or anti-p85 (**C**) antibodies of Rai-specific immunoprecipitates from post-nuclear supernatants of the Rai-expressing JSL1 transfectant activated with anti-CD3 mAb for 1.5 min. Control blots of the stripped filters are shown below.
(TIF)
