# Supplementary figures and images for: The Shc Family Protein Adaptor, Rai, Negatively Regulates T Cell Antigen Receptor Signaling by Inhibiting ZAP-70 Recruitment and Activation
Source: PLoS One. 2011 Dec 29;6(12):e29899. doi: 10.1371/journal.pone.0029899 (PMC3248456; doi:10.1371/journal.pone.0029899)

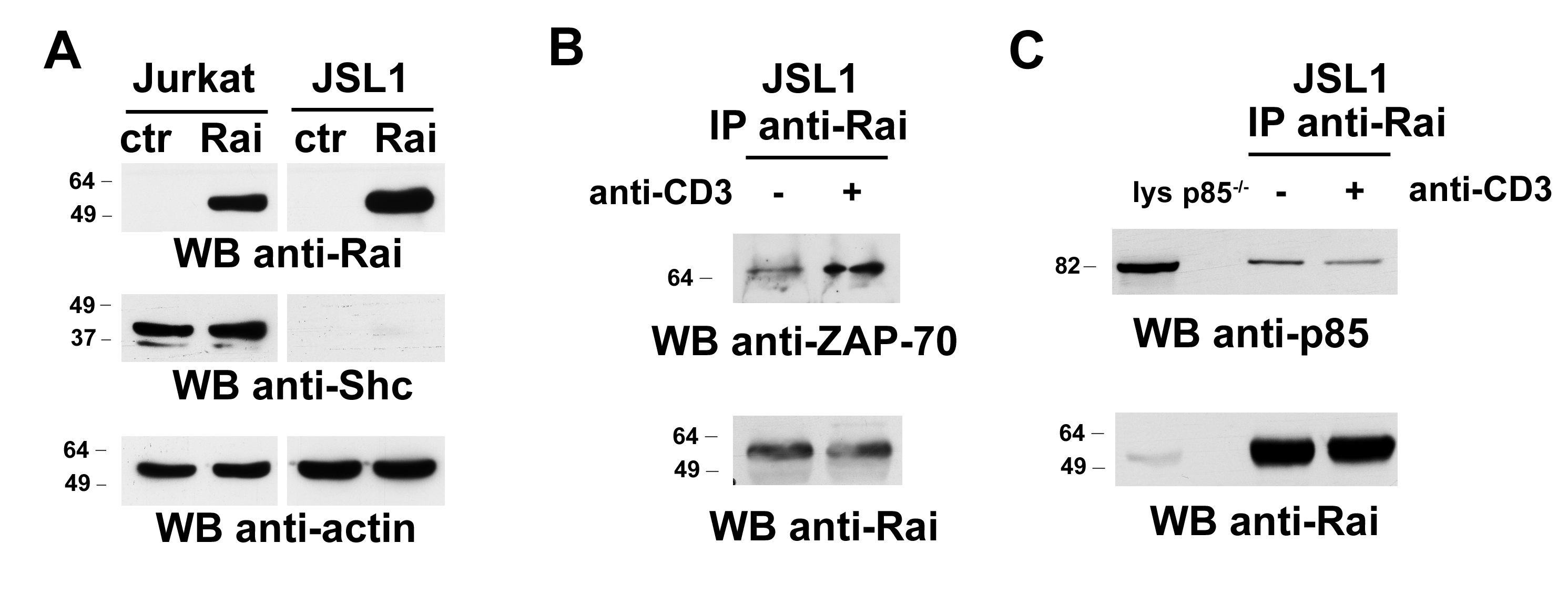

Supplement: Figure S1 — A. Immunoblot analysis with anti-Rai, anti-Shc and anti-actin antibodies of postnuclear supernatants from Jurkat and JSL1 cells stably transfected with either empty vector (ctr) or the same vector encoding the p52 kDa isoform of Rai. B,C. Immunoblot analysis with anti-ZAP-70 (B) or anti-p85 (C) antibodies of Rai-specific immunoprecipitates from post-nuclear supernatants of the Rai-expressing JSL1 transfectant activated with anti-CD3 mAb for 1.5 min. Control blots of the stripped filters are shown below. (TIF) [file pone.0029899.s001.tif]
